# Supplementary material for: End Sequence Analysis Toolkit (ESAT) expands the extractable information from single-cell RNA-seq data
Source: Genome Res. 2016 Oct;26(10):1397–410. doi: 10.1101/gr.207902.116 (PMC5052061; doi:10.1101/gr.207902.116)
Supplement: Supplemental Material [file supp_gr.207902.116_Supp_Materials.tar.gz › ESAT-esat_v0.1/doc/html/dir_22b31a72d531d083c58d6319adb0b504.html]

ESAT: java/broad/projection/gsa Directory Reference


ESAT


- Main Page
- Related Pages
- Packages
- Classes
- Files

All Classes Namespaces Files Functions Variables Enumerator Pages

- java
- broad
- projection
- gsa

gsa Directory Reference

Directory dependency graph for gsa:

|  |  |
| --- | --- |
| Files | |
| file | DefineGeneSignatures.java |
|  | |
| file | ExtractGenes.java |
|  | |
| file | GeneSetEnrichment.java |
|  | |
| file | GSAScoring.java |
|  | |
| file | GsaUtils.java |
|  | |


---

Generated on Fri Aug 15 2014 15:11:15 for ESAT by  

 1.8.7
